# Dietary Supplements among Children Ages 0–3 Years in Poland—Are They Necessary?

**DOI:** 10.3390/foods12010016

**Published:** 2022-12-21

**Authors:** Dagmara Woźniak, Juliusz Przysławski, Michalina Banaszak, Sławomira Drzymała-Czyż

**Affiliations:** 1 Department of Bromatology, Poznan University of Medical Sciences, Rokietnicka 3, 60-806 Poznan, Poland; 2 Poznan University of Medical Sciences Doctoral School, Poznan University of Medical Sciences, Bukowska 70, 60-812 Poznan, Poland

**Keywords:** vitamin D, omega-3 acids, nutritional programming, contamination

## Abstract

(1) Background: One of the ways to prevent nutritional deficiencies may be supplementation. Experts have observed the increased use of dietary supplements, not only in adults but also in children. Considering controversies among dietary supplements and possible errors in children’s feeding, the goal of our research was to evaluate use and reasons behind supplementation in terms of children’s diet analysis. (2) Methods: Our research involved 507 legal guardians of the youngest children (up to 3 years of age) and was conducted via a questionnaire. (3) Results: 79% of all children received dietary supplements. The analysis of children’s diets showed a need to implement omega-3 acids and vitamin D supplementation, which was very low in children. On the other hand, vitamin C, vitamin B, vitamin A, and copper levels were extremely high. (4) Conclusions: Popularity of dietary supplements in Polish children aged 0–3 years old is an omnipresent issue. Although the reasoning behind administering nutritional supplements to children seems justified, considering the supply of vitamin D and omega-3 fatty acids, it seems justified to increase parents’ knowledge in this regard in terms of the use and means to choose the best supplement possible, as dietary supplementation should always be tailored to individual needs.

## 1. Introduction

During women’s pregnancy and the first 1000 days of a child’s life, nutrition is essential to children’s development and later health in adult life. Either excess or lack of nutrients can permanently program metabolism and increase the risk of health conditions, e.g., being overweight, type 2 diabetes, or hypertension [1,2,3,4,5,6]. According to the WHO, a healthy diet helps protect against malnutrition and its consequences [7]. Furthermore, the United Nations International Children’s Emergency Fund (UNICEF), in the report ‘The State of the World’s Children 2019: Children, food and nutrition’, notes that the problem of “hidden hunger” can be prevented through nutritional education aimed at persuading families, children, and young people to reach for food rich in nutrients [8]. Micronutrient deficiencies should be addressed through diet improvement (better quality or quantity), food fortification, or supplementation [7].

As already mentioned, one of the ways to prevent nutritional deficiencies may be supplementation. In the case of healthy children consuming a traditional diet, global teams of scientists recommend the supplementation of only vitamin D. Still, experts have observed the increased use of dietary supplements, not only in adults, but also in children’s diets [9,10,11,12,13]. A survey conducted in the USA from 2007 to 2010 showed that 31% of children aged 0–19 years (n = 8245) took dietary supplements. Most children took one (86%) or two (10%) dietary supplements in the preceding month [14,15]. According to dietary recommendations for children in Poland, vitamin D (400 IU/24 h) should be given to all infants up to the age of 6 months and 600 IU/24 h for infants aged 6–12 months [16]. In risk groups, vitamin D supplementation is also indicated in children over 1 year of age. Breastfed infants receive long-chain polyunsaturated fatty acids with their mother’s milk and do not need additional supplementation [16]. The current content of DHA in infant formulas should also cover the demand for this ingredient in the case of artificial feeding [16]. DHA supplementation may be beneficial when dietary intake of DHA is insufficient (<100 mg/24 h) [16]. Preventive iron supplementation is not recommended. To prevent bleeding from vitamin K deficiency, each newborn should receive prophylactic vitamin K1 after birth [16]. Dietary recommendations do not include probiotics.

Dietary supplements are complementary elements designed to improve nutritional status or complement a diet with necessary nutrients. They may have preventive or curative influence. For example, dietary supplements rich in vitamin D prevent its deficiency and ensure bone health, and supplements with iron can cure iron deficiency [17]. Supplements may contain amino acids, biological or animal extracts, herbal extracts, vitamins, and minerals [18,19,20,21]. The increasing popularity of dietary supplements is reflected in the market—approximately 4.4% or above of the supplement market is dedicated to children [22,23].

According to Barnes et al., in 2007, the most popular dietary supplements among children were: echinacea (37.2%), fish oil, omega-3 fatty acids, or docosahexaenoic acid (DHA) (30.5%), combination products (17.9%), and flaxseed oil (16.7%) [24,25].

The study update conducted in 2012 showed that these preferences did not change, except for fish oil becoming the most popular, followed by probiotics [24,25]. According to Bailey et al., multivitamins, vitamin C, and calcium are also prevalent [19,20].

Apart from the popularity, there are many reasons for paediatric dietary supplementation. Parents often decide to enhance their children’s diets due to a specific condition (chronic infections, attention-deficit hyperactivity disorder, cancer, asthma, malnutrition, common cold, and picky eating) [21,26]. The most popular reasons are to improve or maintain good health, supplement the diet, decrease the risk of health problems, or increase immunity [19,20]. The youngest children (below 2 years of age) also receive dietary supplements to relieve some painful symptoms during teething [19,20].

The analysis of children’s nutrition across developed countries brought up some concerning news. According to the Feeding Infants and Toddlers Study (FITS), children who were 6–47 months had intakes above the Tolerable Upper Intake Level (UL) for vitamin A, folate, zinc, and sodium from their diets, whilst intake of zinc and vitamin A was above the UL in users of dietary supplements [27]. On the other hand, the Infant Feeding Practices Study II stated that vitamin D intake in children 1–10.5 months was too low according to the recommendation, regardless of infant feeding methods (breastfeeding, mixed-feeding, or formula-feeding) [28].

Considering controversies among dietary supplements and possible errors in children’s feeding, the goal of our research was:

1. To assess the use and characteristics of dietary supplements in children aged 0–3 years

2. Characterize parents giving their children supplements, considering the reasons for their behaviour, their age, place of residents and education

3. Assess the association of supplements’ use and anthropometric parameters

4. Assess the validity and possible indications of using dietary supplements, by analysing children’s diets and identifying their nutritional needs.

## 2. Materials and Methods

### 2.1. Participants

Our research involved 507 legal guardians of the youngest children (up to 3 years of age). The parents were recruited at five Paediatric Outpatient Clinics in Poland in 2019–2022. The anthropometric parameters (children’s weight and height) were measured by a paediatrician nurse.

Parents of 507 children completed the study. Although we enrolled parents of 525 children, parents of 18 could not appropriately note the nutrition diary of their children.

Inclusion and exclusion criteria were as described previously [29].

### 2.2. Ethical Consideration

All legal guardians of the children involved in the study gave their informed written consent for participation in the research. The research adhered to the tenets of the revised Declaration of Helsinki. The protocol of the investigation was approved by the Ethical Committee of Poznań University of Medical Sciences, Poznań, Poland (decision no 723/19).

### 2.3. Dietary Intake

Parents provided their children’s menu information over three days, including meals, snacks, and fluids. Parents also provided information on their children’s dietary supplements (detailed information on the type of supplement, the dose used, how often it is taken, etc.). Breastfeeding was assessed. Fortified packaged foods and infant formulas were also included. Infants’ menus also contained information on breastfeeding and formula milk. Parents accounted for the amount of milk drunk by the infants. Breastfeeding and formula feeding were taken into account in calculating quantitative and qualitative dietary intake. The eating schedule had to include one weekend day. Before submitting the eating diary, parents were trained by a certified dietitian and instructed on how to properly complete the food diary, e.g., using the page to help determine the proper food portions (www.ilewazy.pl, available online, accessed on 11 August 2009). An e-mail and telephone number to the dietician were also available in case of any questions. Children’s diets were analyzed using Dietetyk 2015 (Jumar Software, Poznan, Poland) and the NutritionData.com database. According to Polish nutritional standards, the average daily macronutrient intake of children was calculated and compared to the recommended dietary allowance (RDA) [29,30]. Dietary supplementation was also included in the assessment. According to information provided by supplement producers, the dietician accounted for them in dietary analysis. Finally, the analysis of children’s diets considered the children’s overall intake of nutrients from the diet and the analysis of the diets of children who took individual dietary supplements (vitamin D, C, multivitamins, etc.).

### 2.4. Statistical Analyses

All statistical analyses were performed in two different statistical software MedCalc 19.6 (MedCalc Software, 1993–2020) and GraphPadPrism 5.01 (GraphPad Software, Inc., La Jolla, CA, USA). The normality of the data distribution was determined using the Shapiro–Wilk test. For non-normal variables, medians and 1st–3rd quartiles were given. Differences between groups were assessed using the Mann–Whitney test. A *p*-value of <0.05 was considered to indicate significance.

The nutritional status of the children was evaluated based on the standardized Z-score for weight concerning the cut-off points established by the WHO [31].

## 3. Results

### 3.1. Participants

Most respondents lived in a city with fewer than 100 thousand residents (60%) and had higher education (76%). Table 1 shows the representation of the groups.

### 3.2. Assessment of the Prevalence of Use and the Characteristics of Dietary Supplements Taken in Children Aged 0–3 Years

A total of 79% of all children (n = 400) received dietary supplements. In the group of children aged <6 months, 80% took dietary supplements (n = 162); in the group aged 6–12 months—78% (n = 151), and over 12 months—78% (n = 87). Details are presented in Table 2. A total of 15% of children (n = 60) took different supplements (e.g., for constipation, teething).

Out of 400 children who received dietary supplements, 86% (n = 344) took only one dietary supplement, 12% (n = 48)—two dietary supplements and 2%-(n = 8)—three. The most popular forms were gummy bears (90%, n = 360) and soluble tablets (9%, n = 36).

### 3.3. Characteristics of Parents Giving Their Children Supplements, Considering the Reasons for Their Behavior

While 47% (n = 188) of children received dietary supplements due to a paediatrician’s advice, 31% (n =124) did so on the advice of a family member or family friend, and 22% (n = 88) because their parents were susceptible to marketing and advertising of dietary supplements. Additionally, 60% (n = 240) of parents used dietary supplements to improve their children’s hea lth and immunity, 23% to improve their diets (n = 92), 15% (n = 60) to prevent health problems, and 2% (n = 8) were not able to explain their reasons. Table 3 describes the characteristics of the groups in terms of dietary supplement use.

### 3.4. Assessment of the Association between Supplements’ Use and Anthropometric Parameters

The body weight and height comparison between the groups did not reveal any significant differences (Table 4).

### 3.5. Assessment of the Validity of Using Dietary Supplements by Analyzing Children’s Diets and Identifying Their Nutritional Needs

In the group of children aged < 6 months, 86% were breastfed (89% of dietary supplements users—DS and 76% of non-users—NDS). In the group of children aged 6–12 months—only 42% (DS—44%, NDS—33%), and over 1 year—12% (DS—12%, NDS—23%). Polish experts on children’s nutrition recommend breastfeeding for at least 6 months [15]. Regarding modified milk, 22% of children aged < 6 months received formula (sometimes when being also breastfed; DS—23%, NDS—17%), 72% of children aged 6–12 months (DS—75%, NDS—62%), and 22% of children over 1 year (DS—24%, NDS—30%). We did not find any significant differences between breastfeeding and administering dietary supplements. The analysis of children’s diets showed a need to implement omega-3 acids and vitamin D supplementation, which was very low in children (Table 5). On the other hand, vitamin B, vitamin C, vitamin A, and copper levels were extremely high (Table 5). We did not include dietary supplementation in the evaluation.

Children taking vitamin D supplements (n = 324) had significantly higher medium vitamin D intake at the level of 78.96 %RDA (56.12–98.45; *p* < 0.001). The same situation refers to children taking omega-3 supplements (n = 84)—67.09% RDA (45.28–89.02, *p* < 0.001). When it comes to children taking only vitamin C (n = 92)—their vitamin C intake was 236.67 %RDA (170.34–360.53; *p* = 0.760), vitamin K (n = 40)—115.68% RDA (94.21—136.05, *p* = 0.687). Children’s diets enriched in probiotics did not differ significantly regarding vitamins and micronutrient content.

## 4. Discussion

To the authors’ knowledge, this is one of the first studies to evaluate the use of dietary supplements in Polish children at such a young age (up to 3 years old). As Piekara et al. stated, the use of dietary supplements in children is not well-researched and evaluated [23,32]. With this research, we aimed to fill in that gap.

In this study, 79% of children received dietary supplements. In Piekara et al., 40% of 532 Polish parents took dietary supplements themselves, and 54.89% administered them to their children. The use of dietary supplements was also similar. Parents most often chose probiotics and prebiotics (82.53%), vitamin D (74.32%), vitamin C (50.68%), and cod-liver oil (45.89%) [23].

The reasoning behind using dietary supplements in children is very similar in different studies [19,20]. Parents are generally concerned about their children’s health and diets, aiming to improve their condition and protect them from health issues. Respondents in Piekara’s research on Polish parents mentioned similar reasons: wanting to boost a child’s immune system, support during antibiotic therapy or the treatment of infections, introducing vitamins and minerals to the diets [23].

Although in our study, 47% of parents were driven by the paediatrician’s advice to implement dietary supplements, Bailey’s research showed that only 15% of parents from the USA followed medical specialists’ advice [20], but 85% of parents decided to implement dietary supplements [20]. Although their reasons are understandable, there is a need for proper education for parents, not only on the benefits but also the dangers of dietary supplements. Unreasonable choice, dose, and frequency of supplements may affect children’s health [33]. This may be a concern, especially for older children—Bailey’s study proved that parents of children < 2 years old are more likely to stick to the physicians’ advice rather than deciding on their own. According to Piekara’s study, Polish parents are more responsible: 81.51% of them followed the paediatrician’s guidelines to administer supplements. They believed that the decision to administer dietary supplements ought to be made with a doctor (40.23%), or with a doctor or a pharmacist (40.79%) [23].

Our research did not find significant differences between children’s weight and height regarding the reception of dietary supplements. However, according to Bailey et al., the use of dietary supplements was inversely related to weight status [20]. Some studies on children also linked the use of nutritional supplements with more physical activity and care for the body and nutrition [34,35,36].

In our study, 25% of children took multivitamins. In Bailey’s survey, the number of those recipients was far greater, as almost 90% of children who took dietary supplements received multivitamins [20]. The authors stated that the most popular multivitamin products used by children provided > 50% of the daily value for vitamins (A, C, D, E, B2, B6, B12, and folic acid) and minerals (iron, iodine, zinc, and copper) [19,20]. Our analysis of children’s diets showed that the consumed levels of vitamins A, C, B, and copper are very high (over 100% RDA). Unreasonable use of multivitamin supplements may result in exceeding vitamins tolerable upper intake level (UL) [23,37,38]. When it comes to vitamins soluble in water, the excess will be removed from the body with urine. Still, for example, vitamin A is fat-soluble, and its overdose may have severe consequences. Moreover, high supply of some micronutrients—e.g., vitamin D, vitamin A, zinc or iron [23,39,40,41]—may have a synergistic or antagonistic influence on other ingredients in food. Vitamin E and A and vitamin C were found to affect antagonistically on each other in combination with multivitamin supplements [42].

Moreover, vitamin C is commonly treated as an easy way to treat the common cold and is omnipresent, especially during the flu season (from September to February/March). It is worth remembering that high intakes of vitamin C in adults may result in an increased risk of kidney stones [43]. In children, there are not many publications indicating the unambiguous effect of supplementation with too high doses of vitamin C. Nevertheless, because vitamin C is omnipresent in healthy children’s diets in developed countries, additional supplementation is not recommended [44].

In our research, other popular dietary supplements were vitamin D and omega-3 fatty acids. Omega-3 fatty acids are crucial in children’s development, especially regarding immunity and brain development. Omega-3 fatty acid deficiency in infancy is associated with reduced birth rate, childhood stunting, and increased risk of infant mortality [1,45,46]. According to research, supplementing the infant diet with omega-3 fatty acids results in better infant cognitive and visual development. Moreover, full-term infants supplemented with DHA and AA (arachidonic acid) had better sweep visual-evoked potential acuity at several time points, which extended up to 1 year of age [47,48,49]. According to Foiles, healthy, full-term infants fed with AA and DHA-supplemented formula had a reduction in allergic reactions [50].

Vitamin D is crucial for immune development and bone mineralization. Multiple research emphasises the importance of vitamin D in children’s proper development [51,52,53,54,55,56,57,58,59,60,61,62,63,64,65]. The prevalence of vitamin D deficiency in infants and toddlers is estimated to be between 40–50% in the United States [66]. Moreover, recent, extensive dietary analyses have found that about 75% of children aged 6–23.9 months is below the adequate intake—AI (children less than 12 months) or the estimated average requirement—EAR (children 12–23.9 months) for vitamin D [67]. Vitamin D deficiency is an omnipresent issue in the paediatric population. Hence, as mentioned, supplementation with this vitamin is recommended. In Amaro-Riviera et al., the authors draw attention to the fact that vitamin D deficiency can be partly corrected using appropriate dietary supplements [68]. However, Bailey’s research proved that, even among children who took supplements, more than a third of them aged 2–18 years were unable to meet vitamin D recommendations [19,20]. On the other hand, dietary supplement use resulted in higher prevalence of usual intakes above the UL for e.g., iron, zinc, folic acid, and vitamins A and C [19,20].

The great inclusion of products dedicated to children, as well as formula and fortified foods, has an influence on nutrient intake in children’s diets, especially regarding vitamin D and DHA acid [69]. The quality of children’s diets may be connected to different factors, e.g., mother’s knowledge [2], the popularity of commercials, and the family’s eating habits [70]. It is important to remember that improving diet should be one’s first choice in curing deficiencies. Parental knowledge and support of health professionals are also crucial [2].

From the analysis of children’s diets, we can conclude that there is a risk of exceeding upper levels for some ingredients by using dietary supplements, especially when it comes to vitamin A, C, E, B1, B2, B6, and B12. It is worth mentioning the difference between the diets’ qualities between children taking and not taking multivitamins. Children who took multivitamins regularly had a less varied diet, especially regarding vegetables, fruits, vegetable oils, and dairy. In their diets, there were a lot of children’s dedicated products, such as ready-to-eat dinners, snacks, and biscuits. Proper nutritional education should be provided not only to parents but also to health specialists to improve children’s diets in natural nutrients. Moreover, the analysis of children’s diets showed that even with vitamin D and omega-3 supplementation, vitamin D and omega-3 acids demand is still not fulfilled. Vitamin D supplementation, although the most common, is carried out often carelessly. Although it is recommended for children over 6 months of age to supply 800IU per day, parents stick to the dose of 400IU or administer the vitamin every few days. Our results align with experts’ guidelines—reasonable vitamin D and omega-3 supplementation should be considered for children aged 0–3 years old. Their diets should be improved with products naturally rich in those ingredients (fish, dairy, vegetable oils).

In Poland, the Chief Sanitary Inspectorate (pol. Główny Inspektorat Sanitarny—GIS) is the regulatory authority responsible for approving trading admission for dietary supplements, as they are defined as foodstuffs [38]. Thus, introducing a supplement to the market is not challenging. To give their approval, GIS needs the basic information on the product’s name, composition and producer. Supplements negatively evaluated by the European Medicines Agency—EMA, or the US Food and Drug Administration (FDA) can still be sold in Poland [38,71]. 

Thus, it is essential to remember that dietary supplements may benefit human health, but not without some dangers. Despite the perceived risks, our study has not examined the quality of parental supplements. According to Ernst et al., physicians found some serious adverse events in children caused by herbal dietary supplements, including allergies or coma [72], haemolysis and liver failure. Although in our study, parents did not administer herbal supplements to their children, it is worth noticing that the FDA linked homeopathic teething products with more than 400 adverse events (AE) in infants and children. Some of those events were tremors, fever, seizures, vomiting, lethargy, and even death (10 cases) [73]. The FDA warned against using these teething products. However, some damage cannot be reversed.

Another essential matter is possibly adulterating dietary supplements with heavy metals, e.g., lead, mercury, corticosteroids, and pharmaceuticals. Even labelling and information placed on the label may be misleading, with some of the products lacking the ingredients, having different levels than those declared, or differing in terms of the ingredients’ quality and quantity [74,75,76].

Elliot conducted a study to examine the dietary supplement content in the Canadian market. After evaluating 80 different supplements (65% of which were multivitamins), the median dose of ingredients was higher than AI recommendations for vitamins A, B6, B12, and C, B1, B2, B5, and biotin [77].

According to the analysis of children’s diets, the levels of simple sugar are close to the maximum allowed intake (<10% of energy), with a significant number of children consuming too many simple sugars. According to a study performed on data from the documentation of representative samples of dietary supplements (n = 315) available on the Polish market [38], 75.24% of the products contained at least one sweetness agent or sweetener. Sucrose was most often used in production. Too many simple sugars in children’s diets may predispose them to being overweight and to obesity, type 2 diabetes, and insulin resistance, which is especially dangerous considering the epidemy of obesity [78,79]. A popular dietary supplement for children—lollipops—can be made up of more than 90% sucrose or glucose syrup [80]. This unequivocally speaks against this form of supplement. The authors fully agree with the opinion of experts, which indicates that the consumption of free sugars should be limited. In children ≥ 2 years of age and adolescents should be <5% of total energy intake, and in infants and young children intake of free sugars should be even less [81]. The authors recommend that some changes in legislation should be made in terms of sugar contained in dietary supplements, especially in the era of children’s obesity. Such changes are also proposed by UNICEF in its 2019 report [8].

Since over 80% of Polish parents took advice from a paediatrician, it is crucial to provide good nutrition and supplement knowledge to health specialists. The education should cover the indications of supplement use, the ability to recognize their composition, and possible interactions between supplements and other medications and food. The authors believe that the dietary guidelines should be updated frequently regarding recommended dietary supplementation according to the children’s nutritional status and supplementary trends. The guidelines should protect from supplement overuse and, at the same time, enhance diet improvement. The fact that dietary supplements in Europe are easily distributed in the market for sale is disturbing. Stricter guidelines on products’ composition and required clinical trials should be implemented.

The limitation of the study was the authors’ failure to research the parents’ financial status, professional position, and state of health. According to Bailey, using dietary supplements in children is connected with having a higher family income and private health insurance [19,20]. The majority of our respondents had higher education. However, knowledge was not of primary importance when implementing dietary supplements, which is contrary to the Piekara et al. study, where having a higher degree was crucial in administering nutritional supplements to children [23]. It is worth remembering that the group was randomly selected and that poses a limitation to the conclusions and extrapolating the conclusions to the whole population. Similar research needs to be conducted on the representative group of the population.

## 5. Conclusions

The use of dietary supplements in Polish children aged 0–3 years old is an omnipresent issue. Although the reasoning behind administering nutritional supplements to children seems justified, considering the supply of vitamin D (which is in line with the expert recommendations) and omega-3 fatty acids, it seems justified to increase parents’ knowledge in this regard, as dietary supplementation should always be tailored to individual needs.

Future research should focus on randomized controlled trials in order to develop innovative, safe dietary supplements, as well as an exploration of the actual influence of dietary supplements on enhancing diet and development [21]. Polish market rules and definitions regarding nutritional supplements remain controversial and need improvement.

## Figures and Tables

**Table 1:** Data covering parents’ age, education, and place of residence and children’s age and gender.

Parameters	Value
Age of parents (years) ^1^	30 (28–34)
Place of residence	
Population <100 thousand residents	60% (n = 304)
100–500 thousand residents	27% (n = 137)
>500 thousand residents	13% (n = 66)
Education	
Primary	0% (n = 0)
Secondary	24% (n = 122)
Higher	76% (n = 385)
Age of children	
up to the age of 12 months	40% (n = 203)
12–24 months	38% (n = 193)
25–36 months	22% (n = 111)
Gender of children	
Female	57% (n = 289)
Male	43% (n = 218)

^1^ Value = Median (1st–3rd quartile)/%.

**Table 2:** Data concerning dietary supplement use.

Supplement	Number of Children	Breastfeeding ^a^	Infant Formula ^a^	Children Age	Dose Range	Average Time of Admission
Vitamin D_3_	64% (n = 324)	0–6 m: 57% (n = 115) 6–12 m: 20% (n = 39) >12 m: 7% (n = 8)	0–6 m: 2% (n = 4) 6–12 m: 48% (n = 92) >12 m: 16% (n = 18)	0–6 m: 59% (n = 119) 6–12 m: 68% (n = 131) >12 m: 67% (n = 74)	400–800 IU	>6 months
Vitamin C (L-ascorbic acid)	18% (n = 92)	0–6 m: 6% (n = 12) 6–12 m: 9% (n = 18) >12 m: 2% (n = 2)	0–6 m: 0% (n = 0) 6–12 m: 20% (n = 38) >12 m: 7% (n = 8)	0–6 m: n = 6% (n = 12) 6–12 m: 29% (n = 56) >12 m: 22% (n = 24)	100–250 mg	7–31 days
Vitamin K_2_ MK_7_	8% (n = 40)	0–6 m: 10% (n = 20) 6–12 m: 2% (n = 4) >12 m: 0% (n = 0)	0–6 m: 0.5% (n = 1) 6–12 m: 7% (n = 14) >12 m: 0% (n = 0)	0–6 m: n = 10.5% (n = 21) 6–12 m: 9% (n = 18) >12 m: 1% (n = 1)	6–18 µg	<7 days
Probiotics	24% (n = 120)	0–6 m: 34% (n = 69) 6–12 m: 4% (n = 8) >12 m: 0% (n = 0)	0–6 m: 3% (n = 7) 6–12 m: 18% (n = 34) >12 m: 2% (n = 2)	0–6 m: 37% (n = 76) 6–12 m: 22% (n = 42) >12 m: 2% (n = 2)		
• Lactobacillus rhamnosus GG (ATCC 53103)	20% (n = 100)	0–6 m: 29% (n = 59) 6–12 m: 3% (n = 6) >12 m: 0% (n = 0)	0–6 m: 1% (n = 2) 6–12 m: 17% (n = 33) >12 m: 0% (n = 0)	0–6 m: 30% (n = 61) 6–12 m: 20% (n = 39) >12 m: 0% (n = 0)	5–10 × 10^9^ CFU ^b^	7–31 days
• Lactobacillus rhamnosus GG (ATCC 53103)	4% (n = 20)	0–6 m: 4% (n = 9) 6–12 m: 1% (n = 2) >12 m: 0% (n = 0)	0–6 m: 3% (n = 6) 6–12 m: 0.5% (n = 1) >12 m: 2% (n = 2)	0–6 m: 7% (n = 15) 6–12 m: 1.5% (n = 3) >12 m: 2% (n = 2)	1–2 × 10^9^ CFU ^b^	7–31 days
Multivitamins	20% (n = 100)	0–6 m: 2% (n = 4) 6–12 m: 3% (n = 6) >12 m: 1% (n = 1)	0–6 m: 0% (n = 0) 6–12 m: 9% (n = 18) >12 m: 14% (n = 15)	0–6 m: 2% (n = 4) 6–12 m: 12% (n = 24) >12 m: 65% (n = 72)		
• covering up to 50% of RDA for vitamins	8% (n = 40)	0–6 m: 2% (n = 4) 6–12 m: 3% (n = 6) >12 m: 1% (n = 1)	0–6 m: 0% (n = 0) 6–12 m: 5% (n = 10) >12 m: 4% (n = 4)	0–6 m: 2% (n = 4) 6–12 m: 8% (n = 16) >12 m: 5% (n = 5)	1–2 tablets ^c^	1–6 months
• covering over 50% of RDA for vitamins	12% (n = 60)	0–6 m: 0% (n = 0) 6–12 m: 1% (n = 2) >12 m: 0% (n = 0)	0–6 m: 0% (n = 0) 6–12 m: 3% (n = 6) >12 m: 10% (n = 11)	0–6 m: 0% (n = 0) 6–12 m: 4% (n = 8) >12 m: 60% (n = 67)	1–2 tablets ^d^	1–6 months
Omega-3 acids	17% (n = 84)	0–6 m: 9% (n = 18) 6–12 m: 6% (n = 12) >12 m: 1% (n = 1)	0–6 m: 1.5% (n = 3) 6–12 m: 19% (n = 36) >12 m: 4% (n = 4)	0–6 m: 10.5% (n = 21) 6–12 m: 25% (n = 48) >12 m: 14% (n = 15)	0.4 g DHA + 0.3 g EPA	1–6 months

^a^ Children who were breastfed above 50% of all feeds were accounted as ‘breastfeeding’; children who were fed with infant formula above 50% of all feeds were accounted as ‘infant formula’; a lot of children above 12 months of age were neither breastfed nor fed with infant formula—thus the number of children >12 m in ‘Breastfeeding’ and ‘Infant formula’ may not be even. ^b^ CFU—Colony Forming Unit. ^c^ 1 tablet provides a complex of vitamins A (200 µg, E (2 mg), C (10 mg), D_3_ (2 µg), K_1_ (5 µg), B_1_ (0.2 mg), B_2_ (0.2 mg), B_12_ (0.2 mg), folic acid (60 µg); forms: gummy bears, soluble tablet. ^d^ 1 tablet provides a complex of vitamins A (400 µg), E (5 mg), C (40 mg), D_3_ (5 µg), K_1_ (10 µg), B_1_ (0.5 mg), B_2_ (0.5 mg), B_12_ (0.4 mg), folic acid (100 µg); forms: gummy bears, soluble tablet. Vitamin D3, C, K2MK7, omega-3 supplementation was the most prevalent among children aged 6–12 months, probiotics—among children aged 6–12 months, and multivitamins—above 12 months.

**Table 3:** Data concerning parents’ age, education, and place of residence.

Parameters	Parents GR1	Parents GR2	*p*
Age (years) ^1^	30 (28–34)	30 (28–34.5)	0.786
Place of residence ^1^			0.651
Population <100 thousand residents	61%	56%	
100–500 thousand residents	25%	32%	
>500 thousand residents	14%	12%	
Education ^1^			0.755
Primary	0%	4%	
Secondary	25%	20%	
Higher	75%	76%	

GR1—children receiving dietary supplements; GR2—children not receiving dietary supplements; value = Median (1st–3rd quartile)/%; ^1^ Mann–Whitney test.

**Table 4:** Body weight and height deviations in the study groups.

Clinical Parameters	GR1 (n = 400)	GR2 (n = 107)	*p*
	Median (1st–3rd quartile)		
Z-score for weight ^1^	0.330 (−0.818–1.237)	0.112 (−0.705–0.876)	0.640
Z-score for height ^1^	0.777 (0.259–1.657)	−0.101 (−0.642–0.936)	0.156

GR1—children receiving dietary supplements; GR2—children not receiving dietary supplements; ^1^ Mann–Whitney test.

**Table 5:** Dietary intake in children—general dietary intake and dietary intake taking into consideration dietary supplements.

Dietary Intake (% RDA/AI *)	Median (1st–3rd Quartile)		
	General Dietary Intake (n = 507)	Multivitamins ^a^ (n = 40)	Multivitamins ^b^ (n = 60)
Energy	120.48 (109.09–132.12)	114.36 (108.12–124.08)	117.68 (107.92–128.65)
Proteins	285.51 (221.74–325.64)	265.02 (210.04–289.37)	256.17 (211.34–297.56)
Fats	109.69 (88.12–129.56)	98.46 (87.24–112.38)	103.57 (97.50–115.43)
Omega-3 acids	24.52 (10.38–38.43)	26.54 (13.23–43.50)	28.60 (12.40–45.31)
Carbohydrates	112.38 (97.50–124.59)	111.21 (96.40–115.23)	115.50 (93.40–119.32)
Saccharose	94.70 (58.30–158.40)	89.43 (75.30–126.50)	93.30 (65.47–140.30)
Fiber	102.20 (73.90–120.30)	101.10 (72.80–125.40)	100.08 (75.30–123.30)
Potassium *	83.49 (58.35–114.36)	75.30 (45.40–112.33)	86.40 (56.34–110.20)
Sodium *	87.40 (63.01–108.24)	76.42 (59.30–89.60)	74.50 (58.83–84.37)
Calcium	86.01 (73.65–125.48)	85.20 (72.30–110.34)	83.09 (72.10–109.10)
Phosphorus	142.30 (94.61–163.65)	134.10 (88.20–150.20)	149.80 (95.13–169.90)
Magnesium	166.35 (127.50–233.96)	150.55 (115.30–212.38)	168.40 (123.45–235.60)
Iron	87.11 (60.20–124.36)	88.90 (65.20–126.40)	73.45 (56.70–115.60)
Zinc	142.67 (91.73–193.33)	143.20 (90.30–175.20)	136.95 (87.47–160.10)
Copper	175.67 (125.33–266.54)	180.67 (115.20–273.78)	183.40 (115.30–283.14)
**Vitamin A**	**165.44 (123.59–264.49) ^d^**	**205.30 (160.90–290.50) ^d,e^**	**235.50 (175.45–305.28) ^d,e^**
Vitamin D *	**28.11 (12.30–101.86) ^d,e^**	**56.25 (28.30–115.85) ^e^**	**67.80 (30.67–118.90) ^d,e^**
Vitamin E *	**113.10 (83.42–149.93) ^e^**	115.37 (89.30–135.60)	**146.30 (90.35–160.89) ^e^**
**Vitamin C**	**213.43 (164.95–334.69) ^d^**	**257.67 (170.50–380.90) ^d^**	**278.56 (181.30–400.75) ^d^**
Vitamin K *	110.63 (89.23–128.14)	115.78 (93.20–135.40)	121.45 (96.20–138.50)
**Vitamin B1**	**153.81 (103.21–178.80) ^e^**	**176.20 (105.60–190.45) ^d,e^**	**185.06 (113.20–205.45) ^d,e^**
**Vitamin B2**	**208.39 (165.83–273.51) ^e^**	**256.86 (180.90–290.30) ^e^**	**273.40 (178.20–305.12) ^e^**
Niacin	115.83 (75.03–176.47)	110.80 (70.80–165.47)	108.56 (73.21–160.45)
Vitamin B6	208.30 (131.76–316.34)	207.50 (120.90–315.10)	210.87 (125.45–320.08)
Vitamin B12	215.50 (126.56–297.61)	230.45 (130.90–305.20)	245.30 (128.40–310.98)
Folate	119.48 (76.92–169.53)	125.46 (78.20–165.80)	138.23 (80.56–175.90)

* RDA—Recommended dietary allowance according to Polish nutritional standards [30]; for some nutrients—vitamin D, E, sodium, potassium—only AI are established. ^a^ multivitamins covering up to 50% of RDA for vitamins A, E, C, D_3_, K_1_, B_1_, B_2_, B_12_, folic acid. ^b^ multivitamins covering over 50% of RDA for vitamins A, E, C, D_3_, K_1_, B_1_, B_2_, B_12_, folic acid. ^d^ *p* < 0.001. ^e^ *p* < 0.05.

## Data Availability

The data presented in this study are available on request from the corresponding author.
